# The relationship of nomophobia and social media addiction with psychiatric symptoms, self esteem, and perceived parental attitudes in adolescents

**DOI:** 10.1192/j.eurpsy.2025.1156

**Published:** 2025-08-26

**Authors:** S. Kultaş, F. Coşkun, Ö. F. Akça

**Affiliations:** 1 CHILD AND ADOLESCENT PSYCHIATRY, NECMETTIN ERBAKAN UNIVERSITY, KONYA, Türkiye

## Abstract

**Introduction:**

The growing prevalence and impact of digital device usage in everyday life have brought technological addiction to the forefront.Nomophobia and social media addiction are also increasingly significant types of technological addiction among adolescents.

**Objectives:**

The objective of our study is to identify nomophobia and social media addiction in a community sample of adolescents and to examine their associations with psychiatric symptoms, self-esteem and perceived parental attitudes.

**Methods:**

500 adolescents between the ages of 13-18 were included in our study.The entire sample completed a sociodemographic data form, Nomophobia Questionnaire, Social Media Addiction Scale for Adolescents Short Form, Children’s Anxiety and Depression Scale Child Form, Conners-Wells Adolescent Self-Report Scale Short Form, Rosenberg Self-Esteem Scale and Parentıng Style Scale.

**Results:**

%56.6 of the sample had moderate or higher severity nomophobia and %31.4 had social media addiction.In both boys and girls, nomophobia and social media addiction were found to be positively correlated with attention deficit and ADHD index. In addition, in boys, nomophobia and social media addiction were found to be positively correlated with behavioral problems and hyperactivity. In girls, only social media addiction and behavioral problems were positively correlated. In both girls and boys, nomophobia and social media addiction were found to be positively correlated with RCADS subscales and self-esteem scale. In both girls and boys, nomophobia and social media addiction were found to be negatively correlated with acceptance-involvoment and psychological autonomy. In addition, a positive correlation was found between nomophobia and strictness-supervision subscale in girls. As a result of regression analysis: total anxiety was found to predict nomophobia in both girls and boys, total anxiety and obsessive-compulsive disorder were found to predict social media addiction in boys, ADHD index and major depression were found to predict social media addiction in girls. In the path analysis, it was found that behavioral problems and parental attention interacted with each other and predicted social media addiction; social media addiction predicted internalizing disorders both directly and indirectly through self-esteem.

**Conclusions:**

The results of our study explain the relationship between nomophobia and social media addiction and psychiatric symptoms, self-esteem, and perceived parental attitudes. Considering that early detection and intervention of psychiatric disorders reduces the risk of addiction, our study is an important step towards raising awareness about technological addictions.

**Disclosure of Interest:**

None Declared

